# Incidence and risk of hepatic toxicities associated with anaplastic lymphoma kinase inhibitors in the treatment of non-small-cell lung cancer: a systematic review and meta-analysis

**DOI:** 10.18632/oncotarget.23840

**Published:** 2017-12-16

**Authors:** Bing Liu, Maoxi Yuan, Yi Sun, Ziming Cheng, Zaiyong Zhang, Shizheng Hou, Xiangdong Wang, Jingfeng Liu

**Affiliations:** ^1^ Department of Thoracic Surgery, Linyi Central Hospital, Yishui 276400, Shandong Province, China

**Keywords:** ALK TKIs, liver toxicity, lung cancer, meta-analysis

## Abstract

**Background:**

Two anaplastic lymphoma kinase (ALK)-tyrosine kinase inhibitors (-TKIs) have been approved for the treatment of patients with ALK-rearranged (ALK-positive) advanced non-small cell lung cancer (NSCLC). Severe hepatotoxicity has been observed in several clinical studies. We aim to assess the incidence and risk of liver toxicity with these drugs by a systematic review and meta-analysis of clinical trials.

**Materials and Methods:**

The databases of PubMed, Web of Science and abstracts presented at oncology conferences’ proceedings were searched for relevant studies from January 2000 to January 2017. Summary incidence rates, relative risks (RRs), and 95% confidence intervals (CIs) were calculated by using either random effects or fixed effect models.

**Results:**

A total of 1,908 patients from 10 clinical trials were included. The incidences of all-grade aspartate aminotransferase (AST) and alanine transaminase (ALT) elevation were 25.2% (95% CI 17.7–34.7%), and 26.0% (95% CI 17.8–36.3%), respectively. The incidences of high-grade (grade 3 and 4) AST and ALT elevation were 7.0% (95% CI: 5.4–9.0%), and 9.9% (95%CI: 5.6–16.7%), respectively. Sub-group analysis according to ALK-TKIs showed that the incidence of liver toxicities associated with ceritinib was higher than that of crizotinib and alectinib. In comparison with chemotherapy, ALK-TKIs significantly increased the risk of developing all-grade and high-grade AST elevation (RR, 2.30, 95%CI: 1.87–2.83, *p* < 0.001; RR 10.14, 95% CI: 3.9–26.39, *p* < 0.001) and ALT elevation (RR 2.37, 95%CI: 1.97–2.86, *p* < 0.001; RR 7.34, 95% CI: 3.95–13.63, *p* < 0.001), respectively.

**Conclusions:**

The use of ALK-TKIs significantly increases the risk of developing all-grade and high-grade liver toxicities in lung cancer patients.

## INTRODUCTION

Lung cancer is the most common cancer and the leading cancer-related deaths around the worldwide [1]. The majority of lung cancer cases (approximately 80–85%) are diagnosed as non-small-cell lung cancer (NSCLC), including squamous carcinoma, adenocarcinoma and large cell carcinoma [2]. Although platinum-based doublets chemotherapy remains the standard treatment for advanced NSCLC with good performance status, a variety of novel molecular targeted agents have been introduced into clinical practice due to major progress in the understanding of the pathogenesis of NSCLC, which blocking dysregulated signaling pathways [3–5]. signaling pathway, implicating in the cancer cell proliferation and survival, is the first oncogenic drivers to be found in lung cancer [6]. Indeed, published data from clinical trials has shown that EGFR-tyrosine kinase inhibitors (TKIs) are superior to conventional chemotherapy in advanced NSCLC patients presented with EGFR mutation [7–10]. Recently, NSCLC patients harboring an anaplastic lymphoma kinase (ALK) -rearrangement represent the second oncogene addiction to be identified in NSCLC [11, 12]. Rearrangements of the ALK gene are present in in several human cancers and occur in approximately 5% of advanced NSCLC. Specific ALK-TKIs have been developed during the past decade. Currently, two ALK-TKIs, crizotinib and ceritinib, have been approved in many countries worldwide for the treatment of advanced/metastatic ALK-rearranged NSCLC patients [13, 14].

Generally, the common toxicities associated with ALK-TKIs are gastrointestine toxicities including nausea, vomiting and diarrhea, and the severity of these toxicities is mild [15–17]. However, physicians pay more attentions to the incidence and risk of liver toxicities associated with ALK-TKIs when administrating these drugs, although the mechanism of ALK-TKIs-related liver toxicities remains unknown [18]. Current recognition of its risk are generally from single individual clinical trial, but the sample of these studies are small and might have patient selection bias. As a result, we conduct a systematic review and meta-analysis of published data associated with ALK-TKIs to investigate the overall incidence and risk of liver toxicities with the administration of these drugs.

## MATERIALS AND METHODS

### Clinical end point

Three variables were separately considered such as expression of hepatotoxicity: the increase of alanine aminotransferase (ALT) and the increase of aspartate aminotransferase (AST). For each variable, we considered the increase of all grades and grade 3–4 as the main outcomes and the analysis was conducted in order to find a significant difference between the two arms. Adverse events were defined as per version three of the National Cancer Institute’s Common Terminology Criteria for Adverse Events criteria because of its use in the selected trials (NCI-CTC, version 3 or 4; http://ctep.cancer.gov). In the event a study reported high-grade but not low-grade liver toxicities, no assumption of all-grade incidence was made.

### Data sources

We searched the Pubmed (data from Jan 2000 to Jan 2017), Embase (data from Jan 2000 to Jan 2017) and the Cochrane Library electronic databases. Key words were “ALK-TKIs”, “ALK inhibitors”, “crizotinib”, “ceritinib”, “alectinib”, “non-small-cell lung cancer”, “non-small-cell lung carcinoma”, “prospective trials” and “liver toxicities”. The search was limited to prospective clinical trials published in English. We also searched abstracts containing the term “ALK-TKIs” that were presented at the American Society of Clinical Oncology (ASCO) and European Society of Medical Oncology (ESMO) annual meetings from 2004 to 2017 to identify relevant studies. Additionally, we searched the clinical trial registration website (http://www.ClinicalTrials.gov) to obtain information on the registered prospective trials. Each publication was reviewed and in cases of duplicate publication only the most complete, recent, and updated report of the clinical trial was included in the meta-analysis.

### Study selection

Phase I trials were excluded from analyses due to multiple dose level and limited sample sizes, and only prospective phase II/III trials evaluating ALK-TKIs in NSCLC patients with adequate data on liver toxicities were incorporated in the analysis. Clinical trials that met the following criteria were included: (1) prospective phase II or III trials involving NSCLC patients; and (2) available data regarding events or incidence of liver toxicities and sample size. If multiple publications of the same trial were retrieved or if there was a case mix between publications, only the most recent publication (and the most informative) was included.

### Data extraction

Data abstraction was conducted independently by two investigators, and any discrepancy between the reviewers was resolved by consensus. For each study, the following information was extracted: first author’s name, year of publication, trial phase, number of enrolled subjects, treatment arms, number of patients in treatment and controlled groups, median age, median progression-free survival, and adverse outcomes of interest (liver toxicities).

### Statistical analysis

For the calculation of incidence, the number of patients with liver toxicities in ALK-TKIs group and the total number of patients receiving ALK-TKIs were extracted from the selected clinical trials; the proportion of patients with liver toxicities and 95% confidence interval (CI) were derived for each study. To calculate relative risk (RR), patients assigned to ALK-TKIs were compared only with those assigned to control treatment in the same trial. For one study that reported zero events in the treatment or control arm, we applied the classic half-integer correction to calculate the RR and variance [19]. Between-study heterogeneity was estimated using the χ^2^-based Q statistic [20]. Heterogeneity was considered statistically significant when *P*_heterogeneity_ < 0.1. If heterogeneity existed, the pooled estimate calculated based on the random-effects model was reported using the DerSimonian et al method [21]. In the absence of heterogeneity, the pooled estimate calculated based on the fixed-effects model was reported using inverse variance method. A statistical test with a *p*-value less than 0.05 was considered significant. The presence of publication bias was evaluated by using the Begg and Egger tests [22]. The Jadad scale was used to assess the quality of randomized controlled trials based on the reporting of the studies’ methods and results [23]. And we used the Newcastle–Ottawa quality assessment scale to assess the quality of non-comparative (uncontrolled) studies. We selected items that focused on representativeness of study patients, demonstration that the outcome of interest was not present at the start of the study, adequate assessment of outcome, sufficient length of follow-up to allow outcomes to arise, and adequacy of follow-up (Supplementary Table 1). All statistical analyses were performed by using Version 2 of the Comprehensive MetaAnalysis program (Biostat, Englewood, NJ).

## RESULTS

### Eligible studies

**Figure 1:**
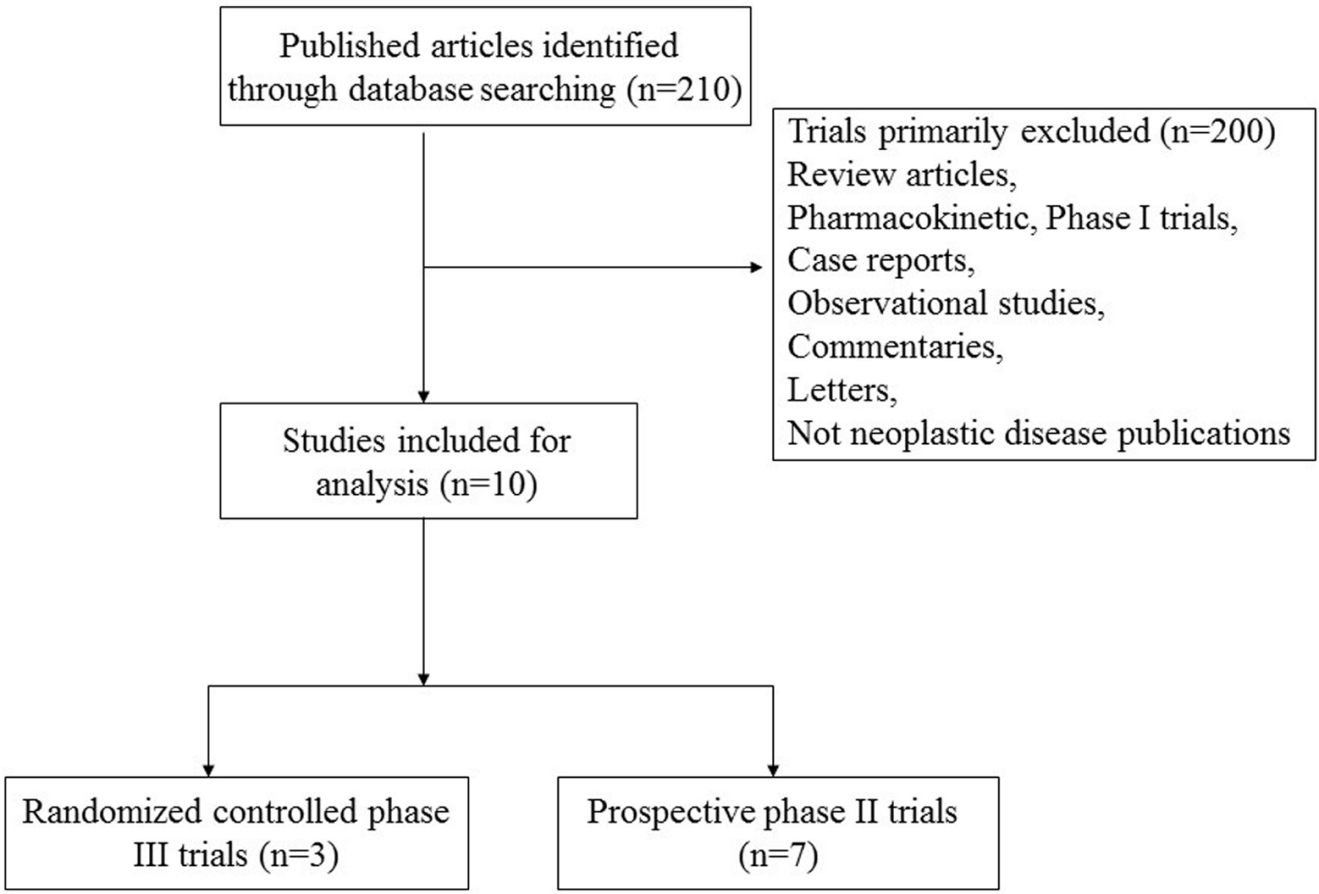
Flow chart of trial selection process in the meta-analysis

A total of 210 records relevant to ALK-TKIs were identified according to the search strategy. The reasons for study exclusion were shown in Figure 1, and we finally selected 10 prospective trials, included 3 phase III [24–26] and 7 phase II trials [27–33] (Table 1). The present study was conducted based on the Preferred Reporting Items for Systematic review and Meta-Analysis (PRISMA) statement (Supplementary Table 2) [34]. A total of 1,908 patients were available for the meta-analysis. The characteristics of patients and studies were listed in Table 1. Based on the eligibility criteria of each trial, patients with impaired renal, hepatic or hematological function were excluded. All of the three randomized controlled trials were open-label controlled trials, thus had Jadad score of 3. For seven non-randomized controlled trials, the quality score was also high (≥ 6) according to NOS checklists.

**Table 1:** Baseline characteristics of ten included prospective trials

Authors/ year	Phase	Patients enrolled	Treatment Arm	Median age (y)	Median PFS (m)	Median OS (m)	No. for analysis
Kwak E.L. et al/2010 [27]	Expansion cohort	82	Crizotinib 250 mg bid po	51	NR	NR	82
Camidge D.R. et al/2012 [28]	Expansion cohort	149	Crizotinib 250 mg bid po	52	9.7	NR	149
Shaw A.T. et al/2013 [24]	III	347	Crizotinib 500 mg bid po	51	7.7	20.3	172
			Chemotherapy	49	3	22.8	171
Shaw A.T. et al/2014a [29]	Expansion cohort	81	Ceritinib 750 mg qd po	53	NR	NR	81
Shaw A.T. et al/2014b [30]	Expansion cohort	50	Crizotinib 250 mg bid po	53	19.2	NR	50
Solomon B.J. et al/2014 [25]	III	343	Crizotinib 500 mg bid po	52	10.9	NR	171
			Chemotherapy	54	7	NR	169
Shaw A.T. et al/2016 [33]	II	87	Alectinib 600 mg bid po	54	NR	NR	87
Kim D.W. et al/2016 [31]	Expansion cohort	255	Ceritinib 750 mg qd po	NR	NR	NR	255
Ou S.H. et al/2016 [32]	II	138	Alectinib 600 mg bid po	52	8.9	NR	138
Soria J.C. et al/2017 [26]	III	376	Ceritinib 750 mg qd po	55	16.6	NR	189
			Chemotherapy	54	8.1	NR	175

Abbreviations: PFS, progression-free survival; OS, overall survival; NR, not reported

### ALT increase

**Figure 2:**
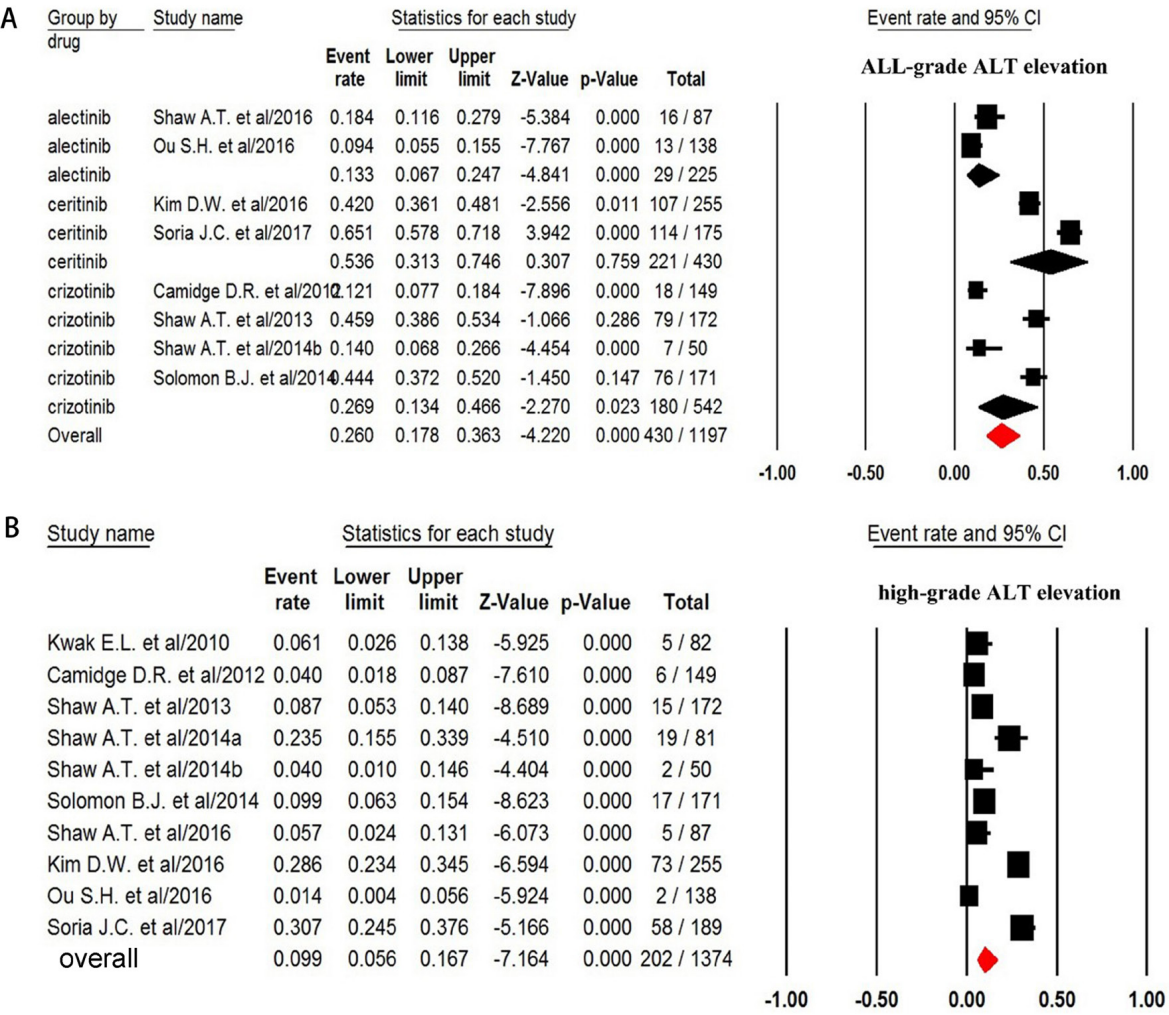
Forest plot for meta-analysis of incidence of all-grade and high-grade ALT elevation in NSCLC patients assigned ALK-TKIs

**Figure 3:**
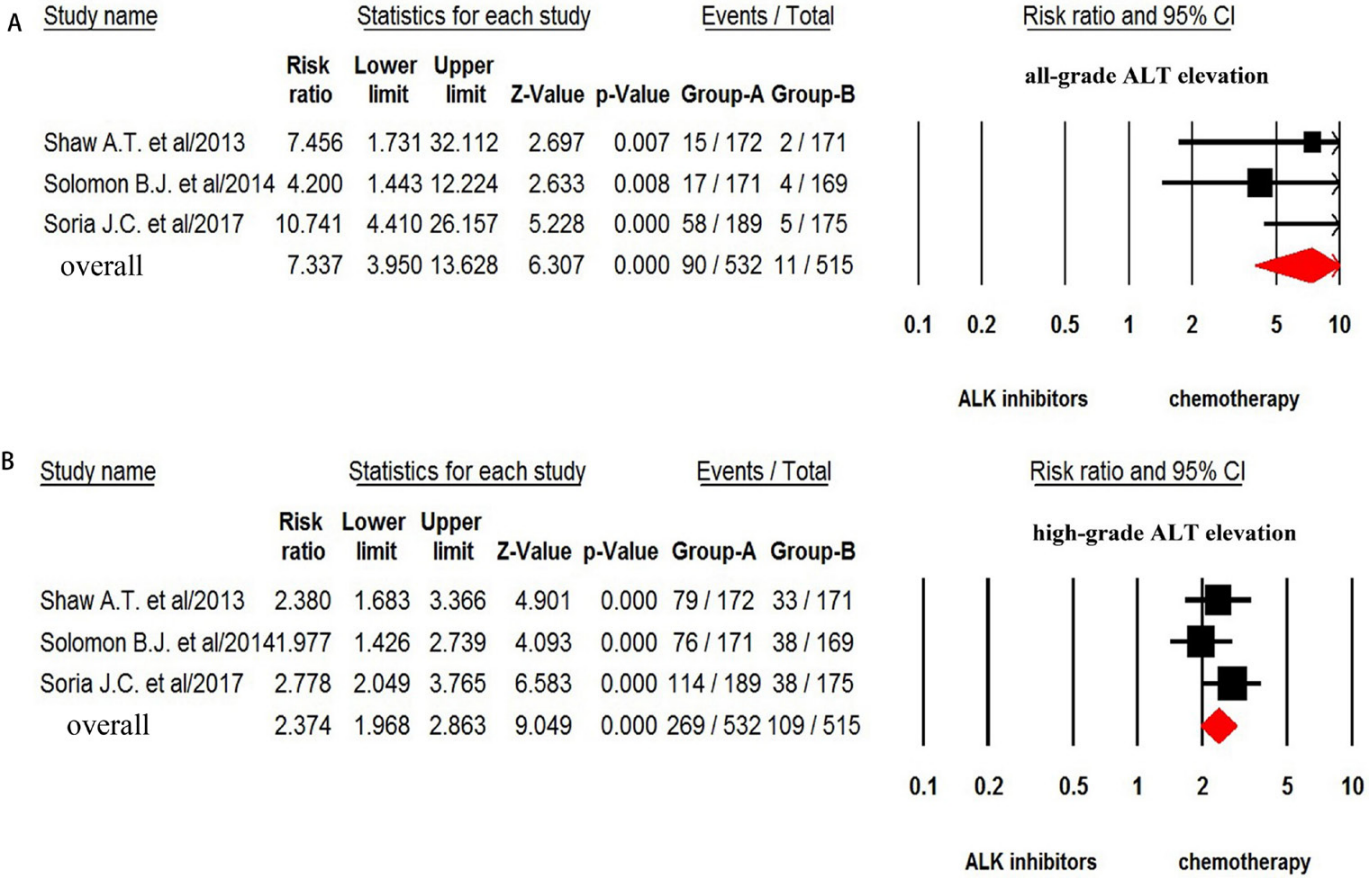
Relative risk of ALK-TKIs-associated all-grade and high-grade ALT elevation versus control from randomized controlled trials

For the overall incidence analysis, only patients received ALK-TKIs alone were included. Thus, a total of 1,197 patients from ten trials were included in the analysis: all-grade ALT increase was reported in 430 out of 1,197 patients with a pooled incidence of 26.0% (95% CI: 17.8–36.3%, Figure 2A).We then conducted sub-group analysis to investigate the incidence difference among different ALK-TKIs and found that the incidence of ALT associated with ceritinib (53.6%, 95%CI: 31.3–74.6%) was significantly higher than that of alectinib (13.3%, 95%CI: 6.7–24.7%) and crizotinib (26.9%, 95%CI: 13.4–46.6%). The RR (fixed effect) to develop any grade of ALT increase was 3.79 (95% CI, 2.89–4.98; *p* < 0.001) in advanced NSCLC patients received ALK-TKIs in comparison with chemotherapy (*Q* = 4.26, *p* = 0.12; *I*^2^ = 53.1%) (Figure 3A).

The incidence and risk of high-grade (grade 3–4) ALT increase was assessed in 1,374 patients, and the pooled incidence of high grade ALT increase was 9.9% (95% CI, 5.6–16.7%, Figure 2B) for ALK-TKIs. In addition, the use of ALK-TKIs in advanced NSCLC patients significantly increased the risk of developing grade 3–4 of ALT increase with RR of 8.92 (95% CI, 4.67–17.0; *p* < 0.001, Figure 3B). There was no significant heterogeneity in the RR analysis for high-grade ALT increase (*Q* = 2.61; *p* = 0.27; *I*^2^ = 23.4%).

### AST increase

**Figure 4:**
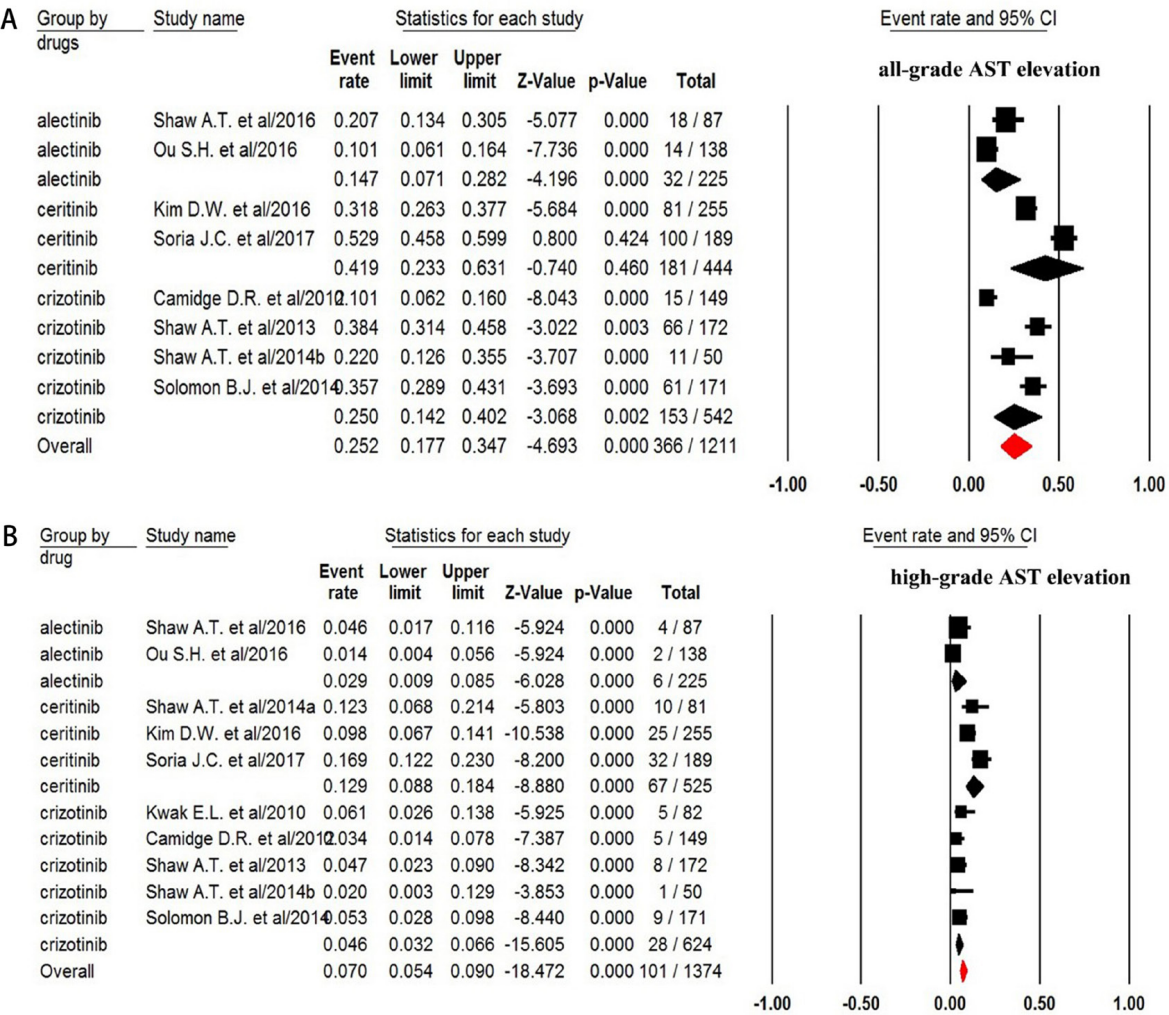
Forest plot for meta-analysis of incidence of all-grade and high-grade AST elevation in NSCLC patients assigned ALK-TKIs

**Figure 5:**
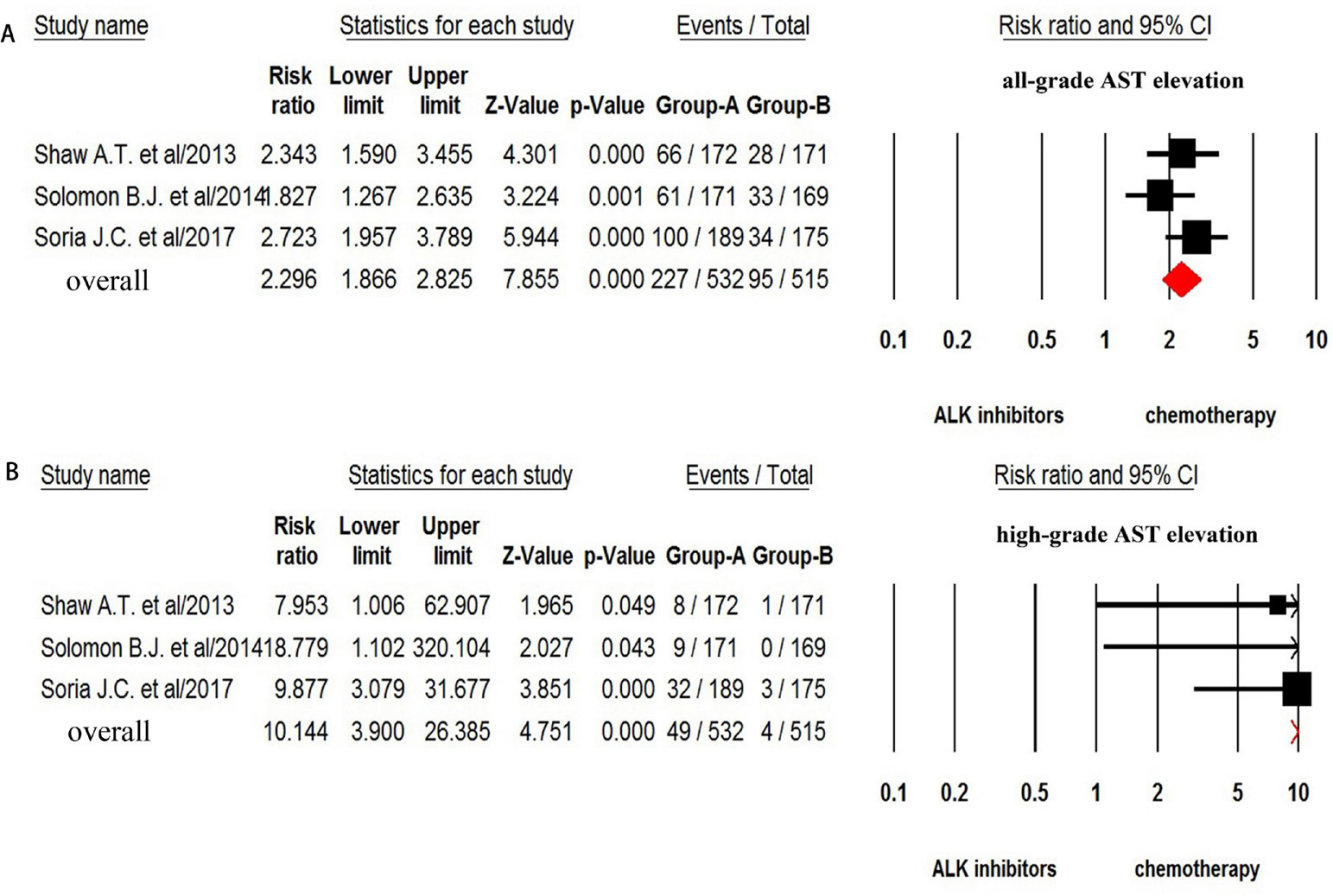
Relative risk of ALK-TKIs-associated all-grade and high-grade AST elevation versus control from randomized controlled trials

For incidence of any grade of AST increase, a total of 1,211 patients were available. All grade of AST increase was reported in 366 out of 1,211 NSCLC patients treated with ALK-TKIs with a pooled incidence of 25.2% (95% CI, 17.7–34.7%, Figure 4A). Sub-group analysis based on the ALT-TKIs demonstrated that the incidence of AST elevation associated with ceritinib (41.9%, 95% CI: 23.3–63.1%) was higher than that of alectinib (14.7%, 95% CI: 7.1–28.2%) and crizotinib (25%, 95% CI: 14.2–34.7%). The risk of developing all-grade AST increase was 3.27 (95% CI, 2.47–4.34; *p* < 0.001, Figure 5A) in advanced NSCLC patients treated with ALK-TKIs in comparison with controls.

The incidence of high-grade (grade 3–4) AST increase was assessed in 1,374 patients and the pooled incidence of high grade of AST increase was 7.0% (95% CI, 5.4–9.0%, Figure 4B) for ALK-TKI. In addition, the risk of developing high-grade AST increase (fix effect) was 11.54 (95% CI, 4.33–30.7; *p* < 0.001, Figure 5B) in advanced NSCLC patients treated with ALK-TKIs in comparison with chemotherapy. There was no significant heterogeneity in the analysis for risk of all grades (*Q* = 4.22%, *p* = 0.12; *I*^2^ = 52.6%) and high-grade (*Q* = 0.23; *p* = 0.89; *I*^2^ = 0%) of AST increase.

### Publication bias

We detected no significant publication biases for all grade of hepatic toxicities by using Begg’s and Egger’s test (*p* = 0.60 and *p* = 0.65 for ALT increase, *p* = 0.60 and *p* = 0.56 for AST increase, respectively). In addition, we did not find significant publication biases for high grades of ALT and AST increase (*p* = 0.60 and *p* = 0.69 for ALT increase, *p* = 0.60 and *p* = 0.81 for AST increase, respectively).

## DISCUSSION

Due to the increased understandings of tumor biology and the signal pathways involved in lung cancer cells proliferation, several novel targeted agents that blocking dysregulated signaling pathways, such as EGFR and vascular endothelial growth factor (VEGF) pathways have been introduced. Although targeted agents are generally well tolerated in lung cancer, severe liver toxicities associated with TKIs have been reported. Indeed, two previously published meta-analyses find that the use of VEGF receptor-tyrosine kinase inhibitors significantly increases the risk of developing liver toxicities [35, 36]. However, the overall incidence and risk of liver toxicities associated with ALK-TKIs remains undetermined. A total of 1908 advanced NSCLC patients from 10 prospective trials are included for analysis, and our study, for the first-time, shows that the use of ALK-TKIs significantly increases the risk of developing liver toxicity. The summary incidences of all-grade ALT and AST increase were 26.0% (95% CI: 17.8–36.3), and 25.2% (95% CI, 17.7–34.7), respectively with 9.9% (95% CI, 5.6–16.7), and 7.0% (95% CI, 5.4–9.0) being high-grade, respectively. Compared to chemotherapy alone, a statistical increase risk of developing all-grade ALT and AST elevations (RR 3.79 and 3.27, respectively) is found in advanced NSCLC patients treated with ALK-TKIs. Additionally, increased risk of developing high-grade ALT and AST elevations are also observed (RR 8.92 and 11.54, respectively) in patients exposed to ALK-TKIs. The findings of the present study will help physicians to fully know the incidence and risk of drug-induced liver toxicities associated with ALK-TKIs in advanced NSCLC patients. Recently, two ALK-TKIs, crizotinib and ceritinib, have been approved for the treatment of advanced ALK-positive NSCLC patients, thus the use of these drugs is anticipated to be increased in anti-cancer treatment and clinical studies. Based on our findings, the following methods might be considered to reduce the potential risk of liver toxicities associated with ALK-TKIs: clinicians should monitor patients during the course of ALK-TKIs treatment and should provide appropriate intervention to reduce morbidity and mortality related to liver damage.

Drug-induced hepatotoxicity is one of the major concerns in clinical practice, because drug-induced liver injury is the most common reason for withdrawal of an approved drug and study terminations. There are several theories for the pathogenesis of drug-induced liver toxicities have been recommended, including immune mediated response, mitochondrial dysfunction and variations in host metabolic response. However, the specific mechanism of ALK-TKIs induced hepatic toxicity remains undetermined, further studies are recommended to address this issues.

Currently, there are no specific guidelines to monitor and manage ALK-TKIs related liver toxicities. According to the experiences from clinical trials, baseline liver function of each patient should be assessed before treatment, and liver function should be monitored every two weeks during the first two months, then monthly and as clinically indicated [18]. For patients with baseline moderate hepatic impairment, a reduced starting dose of ALK-TKIs is recommended. And these drugs should not be used for the treatment of patients with severe hepatic impairment. The majority of susceptible patients will experience liver enzyme elevations in the first few months of drug exposure, and these liver enzyme will return to baseline levels after treatment interruption. For the most of included trials in the present study, dose interruptions or discontinuations are recommended for patients with a raised transaminase levels. For patients with severe aminotransferase elevations, ALK-TKIs should be held until return to pretreatment levels. Then, ALK-TKIs could be resumed at reduced dose.

It should be noted that the present study has several limitations. Firstly, these included trials are performed at various institutions, and there might have potential bias in reported incidences of liver function abnormalities. Second, ALT, AST, and bilirubin elevation represents liver function injury, the sensitivity or specificity of these tests are limited. Thirdly, our study is a meta-analysis of published data, thus individual patient data could not be available, although previous research demonstrate that trial level and patient level meta-analyses yield similar results. Finally, only articles published in English are included for analysis in the present study, which might create some selection bias.

## CONCLUSIONS

In summary, our study has demonstrated that the use of ALK-TKIs significantly increased the incidence and risk of developing all-grade and high-grade hepatotoxicity. Clinicians should clearly recognize the risks and benefits from ALK-TKIs treatment in advanced NSCLC patients, and provide appropriately monitoring of serum transaminases. Additionally, more trials are needed to assess the potential predictive factors for liver toxicities associated with ALK-TKIs treatment in order to avoid premature drug discontinuation.

## SUPPLEMENTARY MATERIALS FIGURES AND TABLES




