# Supplementary material for: Incidence and risk of hepatic toxicities associated with anaplastic lymphoma kinase inhibitors in the treatment of non-small-cell lung cancer: a systematic review and meta-analysis
Source: Oncotarget. 2017 Dec 16;9(10):9480–8. doi: 10.18632/oncotarget.23840 (PMC5823621; doi:10.18632/oncotarget.23840)
Supplement: Supplementary file 1 [file oncotarget-09-9480-s001.pdf]

Incidence and risk of hepatic toxicities associated with anaplastic lymphoma kinase inhibitors in the treatment of non-small-cell lung cancer: a systematic review and meta-analysis

SUPPLEMENTARY MATERIALS

Supplementary Table 1: Newcastle–Ottawa quality assessment scale

**Selection**
1. Representativeness of the exposed cohort
a) Truly representative of the average CRC pancreatic cancer patients in the community (★)
b) Somewhat representative of the average CRC pancreatic cancer patients in the community (★)
c) Selected group of users (eg, nurses, volunteers)
d) No description of the derivation of the cohort
2. Selection of the non-exposed cohort
a) Drawn from the same community as the exposed cohort (★)
b) Drawn from a different source
c) No description of the derivation of the non-exposed cohort
3. Ascertainment of exposure
a) Secure record (eg, surgical records) (★)
b) Structured interview (★)
c) Written self-report
d) No description
4. Demonstration that outcome of interest was not present at start of study
a) Yes (★)
b) No
**Comparability**
1. Comparability of cohorts on the basis of the design or analysis
a) Study controls for metastasis or micro-metastasis (★)
b) Study controls for any additional factor (★)
**Outcome**
1. Assessment of outcome
a) Independent blind assessment (★)
b) Record linkage (★)
c) Self-report
d) No description
2. Was follow-up long enough for outcomes to occur?
a) Yes (2 years) (★)
b) No
3. Adequacy of follow-up of cohorts
a) Complete follow-up – all subjects accounted for (★)
b) Subjects lost to follow-up unlikely to introduce bias – small number lost 25% follow-up, or description of those lost (★)
c) Follow-up rate < 75% and no description of those lost
d) No statement

Supplementary Table 2: PRISMA 2009 Checklist. See Supplementary_Table_2
